# Platelet-Targeted FVIII Gene Therapy Restores Hemostasis and Induces Immune Tolerance for Hemophilia A

**DOI:** 10.3389/fimmu.2020.00964

**Published:** 2020-06-12

**Authors:** Yuanhua Cai, Qizhen Shi

**Affiliations:** ^1^Department of Pediatrics, Medical College of Wisconsin, Milwaukee, WI, United States; ^2^Blood Research Institute, Versiti Wisconsin, Milwaukee, WI, United States; ^3^Children's Research Institute, Children's Wisconsin, Milwaukee, WI, United States; ^4^MACC Fund Research Center, Milwaukee, WI, United States

**Keywords:** platelets, gene therapy, immune tolerance, hemophilia A, factor VIII

## Abstract

Platelets are small anucleated blood components primarily described as playing a fundamental role in hemostasis and thrombosis. Over the last decades, increasing evidence has demonstrated the role of platelets in modulating inflammatory reactions and immune responses. Platelets harbor several specialized organelles: granules, endosomes, lysosomes, and mitochondria that can synthesize proteins with pre-stored mRNAs when needed. While the functions of platelets in the immune response are well-recognized, little is known about the potential role of platelets in immune tolerance. Recent studies demonstrate that platelet-specific FVIII gene therapy can restore hemostasis and induce immune tolerance in hemophilia A mice, even mice with preexisting anti-FVIII immunity. Here, we review the potential mechanisms by which platelet-targeted FVIII gene therapy restores hemostasis in the presence of anti-FVIII inhibitory antibodies and induces immune tolerance in hemophilia A.

## Introduction

Platelets are the second most common type of cells found in blood, with approximately 10^11^ newly produced daily to replenish the old platelets in the body (1, 2). Aged platelets undergo apoptosis and are phagocytosed by scavenger cells in the spleen and liver (3–5). It is increasingly recognized that platelets play fundamental roles not only in hemostasis and thrombosis but also in innate and adaptive immunity. The roles of platelets in the immune response have been extensively reviewed in many papers (6–8), but few studies indicate the role of platelets in immune tolerance. Recent studies that target platelets for gene therapy reveal the potential role of platelets in immune tolerance induction (9, 10).

Platelets are loaded with abundant bioactive proteins and circulate in the blood, serving as both a storage “depot” and trafficking “vehicle” in circulation. Due to these characteristics, platelets may be a unique target for gene therapy of diseases. In the past two decades, several groups have been instrumental in developing novel strategies for hemophilia A gene therapy using platelets as a target (11–20). It has been shown that ectopic expression of factor VIII (FVIII) in platelets directed by either the glycoprotein (GP) Ib or the GPIIb (αIIb) promoter can lead to the storage of FVIII in platelet α-granules and that platelet-derived FVIII can improve hemostasis in hemophilia A mice even in the presence of anti-FVIII inhibitory antibodies (referred to as inhibitors) (13, 15, 17, 21). In addition to achieving hemostatic efficacy, studies have demonstrated that lentivirus-mediated platelet-specific FVIII gene delivery under control of the αIIb promoter (2bF8) to hematopoietic stem cells (HSCs) can induce antigen-specific immune tolerance in hemophilia A mice even with preexisting anti-FVIII immunity (22–24). In this review, we discuss the potential mechanisms of platelet-targeted FVIII expression in restoring hemostasis for hemophilia A in the presence of anti-FVIII inhibitors and inducing immune tolerization after platelet-specific gene therapy.

## Platelets Shield Neoprotein From Being Recognized by the Immune System

Platelets could be an ideal target for gene therapy of hemophilia A as they can store neoprotein FVIII together with its carrier protein von Willebrand factor (VWF) in α-granules and act as delivery vehicles in blood circulation. It has been shown that when FVIII expression is introduced by HSC transduction with 2bF8 lentivirus followed by transplantation, FVIII expression is detected only in platelets, but not in plasma of hemophilia A mice (14, 17, 22, 23). Plasma FVIII is undetectable in 2bF8-transduced recipients even with a platelet-FVIII level as high as 30–35 mU/10^8^ platelets (corresponding to ~60–70% of FVIII in whole blood in normal wild-type C57BL/6 mice) (22, 23). Thus, neoprotein FVIII stored in platelets may avoid direct exposure to the immune system during the normal physiological condition, which may reduce the potential to elicit immune responses against the neoprotein. Indeed, neither inhibitory nor non-inhibitory anti-FVIII antibodies were detected after platelet-specific FVIII gene therapy via 2bF8 lentivirus-mediated bone marrow or HSC transduction followed by transplantation. The efficacy in phenotypic correction and immune tolerance induction was further confirmed through sequential bone marrow transplantations in secondary and tertiary recipients (14, 17, 22, 25).

The effectiveness of platelet-targeted gene therapy has been further confirmed in hemophilia A rats (26) and hemophilia A dogs (27). Shi et al. recently developed a hemophilia A rat model, in which nearly the entire rat FVIII gene is inverted, with a severe spontaneous bleeding phenotype and a high incidence of inhibitor development upon rhFVIII infusion (26). Of note, the severe hemophilic phenotype in hemophilia A rats is fully rescued after platelet-targeted FVIII expression. When platelet-FVIII expression was introduced into hemophilia A rats after transplantation of 2bF8 genetically manipulated bone marrow cells from 2bF8 transgenic rats, the spontaneous bleeding phenotype was rescued with no inhibitor development even though animals were continuously exposed to platelet-FVIII after bone marrow transplantation (26). Using a large animal model, hemophilia A dogs, Du and coworkers demonstrated that 2bF8 lentivirus-mediated HSC transduction followed by transplantation improved hemostasis in hemophilia A dogs and animals were well-tolerized to 2bF8 lentivirus-introduced neoprotein with no detectable inhibitor development in treated animals (27).

In contrast to platelet-specific FVIII expression, it has been shown that targeting FVIII expression to hematopoietic cells under a constitutively active promoter may trigger anti-FVIII immune responses. Wang et al. (20) utilized the intraosseous delivery of a lentiviral vector targeting FVIII to platelets (under the GPIbα promoter, G-F8-LV) and a lentiviral vector with constitutive FVIII expression (under the elongation factor 1α promoter, E-F8-LV). After a FVIII gene transfer by injecting E-F8-LVs into tibias in hemophilia A mice, up to 20% of plasma FVIII activity was detected initially but dropped to undetectable levels within 2–3 months due to the development of FVIII inhibitors. In contrast, in hemophilia A mice that received G-F8-LVs, platelet-derived FVIII was detected and sustained up to 160 days, and a partial phenotypic correction was achieved even with anti-FVIII inhibitors. The difference in efficacy between these two lentiviral treatments may be due to the expression pattern of FVIII. Studies from Kootstra et al. (28) also showed that hemophilia A mice developed anti-FVIII immune responses after non-specific FVIII expression in hematopoietic cells. In their study, FVIII expression was driven by the β-actin promoter (ubiquitous). They showed that all animals developed inhibitors and that transduced cells were eliminated within 4 months after gene therapy.

Besides the promoter, other factors, e.g., protein properties, may also affect the efficacy of gene therapy. Studies by Gangadharan et al. (29) demonstrated that sustained high levels of plasma FVIII were achieved in hemophilia A mice that were preconditioned with either lethal 11 Gy or sub-lethal 5.5 Gy TBI and received Sca-1^+^ or c-kit^+^ cells transduced with porcine FVIII driven by the mouse stem cell virus (MSCV) promoter (MSCV-porcine fVIII) using a retrovirus-mediated gene transfer system. Further studies by Ide et al. (30) demonstrated that sufficient preconditioning is critical for achieving success within MSCV-porcine fVIII/HSC gene therapy in hemophilia A mice. When hemophilia A mice were preconditioned with busulfan or busulfan plus cyclophosphamide followed by transplantation of MSCV-porcine fVIII-transduced Sca-1^+^ cells, transient FVIII expression was obtained in recipients on day 7, but dropped to undetectable on day 14 and afterward due to the development of anti-FVIII inhibitors. When busulfan was supplemented with anti-thymocyte serum (ATS) for preconditioning, sustained plasma FVIII expression was achieved in mice after receiving MSCV- porcine fVIII-transduced Sca-1^+^ cells (30). In lentivirus-mediated platelet-targeted gene therapy, busulfan alone preconditioning is sufficient for achieving sustained therapeutic levels of platelet-FVIII in hemophilia A mice in the non-inhibitor model (17, 23). These studies support that targeting FVIII to platelets is unique in the hemophilia A gene therapy, because FVIII stored in platelets can be better sequestered compared to plasma FVIII as platelet-FVIII will be released together with its carrier protein VWF when it is needed, i.e., at the site of injury where platelets are activated. In addition, sheltering FVIII in platelets can protect the neoprotein from being recognized by the circulating immune cells. Sheltering FVIII in platelets protects FVIII from being inactivated by the circulating FVIII inhibitors.

## The Presence of Protective Protein VWF to FVIII in Platelets is Critical for Optimal Platelet Gene Therapy of Hemophilia A

It is well-known that VWF binds with FVIII non-covalently, which affects the expression and stability of FVIII. FVIII colocalizes with endogenous protein VWF in platelet α-granules when FVIII is targeted to platelets (12, 13). Studies by Shi et al. (31) demonstrated that VWF has a protective effect on FVIII from inhibitor inactivation, and the preformed complex of VWF with FVIII has a greater protective effect on FVIII from anti-FVIII inhibitor inactivation than unbound VWF. When FVIII expression is targeted to platelets, it is stored together with endogenous VWF in a protective compartment, platelet α-granules, where it has an opportunity to form a VWF/FVIII complex. This preformed VWF/FVIII complex will be released locally at the site of injury. Thus, it can reduce inhibitor inactivation of FVIII, achieving hemostatic efficacy.

Further studies by Shi et al. (32) using 2bF8 transgenic mouse models showed that the preformed VWF/FVIII complex is vital for optimal platelet gene therapy of hemophilia A with inhibitors. VWF impacts the expression of platelet-FVIII as well as the hemostasis efficacy. Without VWF, the level of platelet-FVIII significantly decreased, and while hemostatic efficacy was still maintained in hemophilia A mice in the absence of inhibitors, it was limited in the presence of anti-FVIII inhibitors. These results demonstrate that VWF is essential to platelet-targeted gene therapy in hemophilia A with inhibitors. Using 2bF8 transgenic mice in the FVIII knockout background with varying VWF expressions, Shi et al. showed that both platelet-derived VWF and plasma-derived VWF are required for optimal platelet-derived FVIII gene therapy in hemophilia A mice with inhibitors (32).

More evidence indicating the important role of VWF in platelet FVIII gene therapy derives from studies of platelet-targeted FIX gene therapy in hemophilia B mice (33, 34). When FIX expression is targeted to platelets under control of the same platelet-specific αIIb promoter used in the FVIII studies, greater than 90% of FIX is stored in platelets and is releasable upon platelet activation. While the bleeding phenotype is rescued in hemophilia B mice without anti-FIX inhibitors after platelet-targeted FIX gene therapy, the efficacy is nullified in the presence of the anti-FIX inhibitors (33). This differs from platelet-targeted FVIII gene therapy in hemophilia A mice, in which the treatment is effective even in the presence of anti-FVIII inhibitors. We reason that the ineffectiveness of platelet-FIX in the inhibitor model is because there is no protective protein for FIX, so functional FIX activity is rapidly neutralized by circulating anti-FIX inhibitors, once released from activated transduced platelets at the site of injury.

The protective role of VWF in platelet-targeted FVIII gene therapy is not only revealed in its hemostatic function but also in immune responses. To initiate an anti-FVIII immune response, FVIII needs to be internalized by antigen-presenting cells and presented to FVIII-specific CD4 T cells. Studies done by Dasgupta et al. (35) showed that VWF protects FVIII from endocytosis by dendritic cells, which may reduce the immune response to FVIII. VWF can also modulate the repertoire of FVIII-derived peptides on antigen-presenting cells, which may affect the CD4^+^ T cell-mediated anti-FVIII immune response (36). Chen et al. reported that VWF could attenuate FVIII-primed CD4 T cell proliferation in response to rhFVIII restimulation. Their studies showed that VWF could mitigate FVIII-specific memory B cell maturation and anti-FVIII antibody production both *ex vivo* in a memory B cell–based ELISPOT assay and *in vivo* in an immunocompromised FVIII deficient animal model upon rhFVIII restimulation (37). Results from this study support the notion that FVIII stored together with VWF in platelets may be less immunogenic compared to plasma FVIII in a milieu of preexisting anti-FVIII immunity. Indeed, studies by Chen et al. demonstrated that infusion of platelets containing FVIII into hemophilia A mice with pre-existing anti-FVIII immunity did not trigger a memory immune response, but robust memory immune responses were elicited when a similar amount of rhFVIII was infused into plasma (38).

Thus, in our platelet-targeted gene therapy protocol, the association of VWF and FVIII is pivotal for clinical efficacy in hemophilia A with inhibitors. The VWF/FVIII complex protects FVIII from being inactivated by the inhibitors after a burst of VWF/FVIII complex released at the site of injury.

## Proper Preconditioning Before Gene Transfer is Important for Achieving Sustained Platelet-FVIII Expression and Immune Tolerance Induction in Platelet Gene Therapy

Proper preconditioning is essential for immune tolerance induction in our platelet-targeted FVIII gene therapy protocol. Chen et al. (38) reported that the infusion of platelets containing FVIII to hemophilia A mice neither triggered immune responses nor induced immune tolerance to FVIII. However, immune tolerance was induced in mice preconditioned with 6.6 Gy followed by 2bF8 transgenic platelet infusion (38). This could be because the proper preconditioning followed by the introduction of platelet-derived FVIII helps to reconstruct the immune system, especially in the early phases (<8 weeks) of bone marrow reconstitution. It has been shown that ultraviolet (UV) irradiation before antigen immunization could promote antigen-specific immune tolerance through Treg cell induction in mice (39). Studies by Zheng et al. revealed that T cell reconstitution favored Treg differentiation when the mice received sub-lethal irradiation (40). Also, preconditioning can induce large amounts of apoptotic cells, which has been shown to create an immunosuppressive microenvironment (41). All these studies indicate the importance of preconditioning in inducing immune tolerance.

The optimal preconditioning regimen for platelet-FVIII gene therapy to establish immune tolerance while achieving sustained platelet-FVIII expression is more stringent than that used to achieve sustained platelet-FVIII expression alone in unprimed hemophilia A mice. Chen et al. (23) showed that sustained platelet-FVIII expression was achieved, and no anti-FVIII antibodies were detected in 2bF8 lentivirus-transduced recipients preconditioned with either myeloablative 11 Gy TBI, non-myeloablative 6.6 Gy TBI, busulfan, or busulfan plus ATG. Further studies showed that even after rhFVIII immunization, none of the recipients developed inhibitors in the groups preconditioned with an optimized preconditioning regimen, 6.6 Gy TBI or busulfan plus ATG. In contrast, 25 and 40% of the recipients developed inhibitors in the 11 Gy TBI group and the busulfan group, respectively, when they were challenged with the same rhFVIII immunization protocol (23). It's still unclear how preconditioning impacts immune tolerance induction, but studies from our laboratory demonstrate that proper preconditioning is important in our platelet-targeted gene therapy protocol. We speculate that a lethal dose of irradiation (11 Gy TBI) may severely disrupt the intestinal immune system (42), which may impact Treg cell homeostasis in the body. The 11 Gy TBI myeloablative preconditioning may disrupt Treg differentiation, dampening the efficacy of immune tolerance induction after platelet-targeted gene therapy. Thus, proper preconditioning is critical for the effectiveness of platelet-targeted gene therapy in restoring hemostasis and inducing immune tolerance in hemophilia A.

## Peripheral Tolerance is Established After Platelet-Targeted 2bF8 Gene Therapy

Multiple lines of evidence suggest that both primary and secondary anti-FVIII immune responses are CD4 T cell-dependent (43–52). Studies from Chen et al. (23) demonstrated that the immune tolerance induced by 2bF8 lentivirus-mediated gene therapy is CD4 T cell-mediated. Chen et al. found that Treg cells increased in 2bF8-transduced recipients. Using a T cell proliferation assay, they showed that CD4^+^ T cells from rhFVIII-immunized 2bF8 lentivirus-transduced recipients' spleens did not respond to rhFVIII restimulation when co-cultured with dendritic cells from deficient (FVIII^null^) mice. Further studies using a FVIII-specific memory B cell differentiation assay showed that CD4 T cells from FVIII-immunized 2bF8 lentivirus-transduced recipients could not promote memory B cell maturation into antibody-secreting cells, but memory B cells from FVIII-immunized 2bF8-transduced recipients could differentiate into antibody-secreting cells when co-cultured with CD4 T cells isolated from FVIII-primed untransduced FVIII^null^ mice. Further studies showed that immune tolerance is transferable when splenocytes from 2bF8-transduced recipients were infused into naive FVIII^null^ mice (23). Together, data from these studies demonstrate that immune tolerance established in 2bF8 lentivirus-transduced recipients is mediated by the CD4 T cell compartment.

To further investigate how immune tolerance is established after platelet-targeted gene transfer, Luo et al. (10) used the OVA model and utilized the OVA-specific T cell receptor transgenic mice to elucidate the potential mechanisms. They found that antigen-specific CD4 T cells were deleted in peripheral lymphoid organs (spleen and lymph nodes), but not in the thymus, and antigen-specific Treg cells were expanded after platelet-targeted OVA gene transfer. The specific mechanisms related to the OVA expression levels. The deletion of peripheral antigen-specific CD4 T cells was more prominent in mice with a higher level of OVA expression, whereas with a lower OVA level, the increase in antigen-specific Treg cells was dominant. Importantly, even with a lower expression level of ectopic protein, platelet-specific OVA gene transfer could still induce immune tolerance in the unprimed model. The OVA model study reveals that there are dual underlying mechanisms that are responsible for establishing antigen-specific immune tolerance after platelet-targeted gene therapy.

## Conclusion

Platelets play fundamental roles not only in hemostasis and thrombosis but also in innate and adaptive immunity. Data from preclinical trials using animal models have demonstrated that platelet-targeted FVIII gene therapy is effective in treating hemophilia A mice even with inhibitors. Platelet-targeted gene therapy can promote antigen-specific immune tolerance through peripheral tolerance mechanisms. The effectiveness of platelet gene therapy in hemophilia A with inhibitors could be attributed to many pivotal factors, including the shielding of neoprotein by platelets from being recognized by the immune system, the presence of a protective protein VWF in the platelets, and proper preconditioning before gene transfer. In conclusion, platelet-targeted gene therapy is a unique approach for gene therapy of hemophilia A even with inhibitors as it can provide not only therapeutic protein but also induce antigen-specific immune tolerance.

## Author Contributions

YC contributed to manuscript writing. QS conceived the structure of the review and wrote the manuscript.

## Conflict of Interest

The authors declare that the research was conducted in the absence of any commercial or financial relationships that could be construed as a potential conflict of interest.
